# Anatomical, Histological and Histochemical Observations of the Eyelids and Orbital Glands in the Lowland Tapir (*Tapirus terrestris* Linnaeus, 1785) (Perissodactyla: Ceratomorpha)

**DOI:** 10.3390/ani13132081

**Published:** 2023-06-23

**Authors:** Joanna Klećkowska-Nawrot, Karolina Goździewska-Harłajczuk, Marta Kupczyńska, Katarzyna Kaleta-Kuratewicz, Piotr Kuropka, Karolina Barszcz

**Affiliations:** 1 Division of Animal Anatomy, Department of Biostructure and Animal Physiology, Faculty of Veterinary Medicine, Wroclaw University of Environmental and Life Sciences, Kożuchowska 1, 51-631 Wroclaw, Poland; 2 Department of Morphological Sciences, Institute of Veterinary Medicine, Warsaw University of Life Sciences, Nowoursynowska 159, 02-787 Warsaw, Poland; marta_kupczyńska@sggw.edu.pl (M.K.);; 3 Division of Animal Histology, Department of Biostructure and Animal Physiology, Faculty of Veterinary Medicine, Wroclaw University of Environmental and Life Sciences, Norwida 25, 50-375 Wroclaw, Poland; katarzyna.kaleta-kuratewicz@upwr.edu.pl (K.K.-K.); piotr.kuropka@upwr.edu.pl (P.K.)

**Keywords:** adnexa of the eye, deep gland of the third eyelid, lacrimal gland, lowland tapir, superficial gland of the third eyelid, third eyelid, upper and lower eyelids

## Abstract

**Simple Summary:**

The aim of this study was to provide an anatomical and histological description of the eyelids and orbital glands in the lowland tapir (*Tapirus terrestris*). The investigation was conducted on two female adult lowland tapirs from the Wroclaw Zoological Garden (Poland). Our results significantly expand the existing knowledge on the comparative anatomy of chosen accessory organs of the eye in one of the four living families of Tapiridae representatives. We present here the features typical only for tapirs and also some common features for closely related Tapiridae and Rhinocerotidae in terms of the morphology of the upper eyelid, lower eyelid, third eyelid and orbital glands.

**Abstract:**

The lowland tapir is one of four species belonging to the Tapiridae family of the Ceratomorpha suborder, similar to Rhinocerotidae. This study describes anatomy with morphometry, histology (hematoxylin and eosin, Masson-Goldner trichrome, Movat pentachrome, mucicarmine, picro-Mallory trichrome) and histochemistry (PAS, AB pH 1.0, AB pH 2.5; AB pH2.5/PAS and HDI) of the upper and lower eyelids, and superficial gland of the third eyelid with the third eyelid, deep gland of the third eyelid, and lacrimal gland. The aim of the work is to show the features of the above-mentioned structures typical only for Tapiridae, as well as to show the presence of similarities and differences between the families forming the order Perissodactyla. The eyelashes on the upper eyelid were long, while those of the lower eyelid were short and much less prominent. In the upper and lower eyelid sebaceous glands, a characteristic simple alveolar gland producing a mucus-like secretion and poorly developed tarsal glands were observed. The marginal zone of the posterior surface of the eyelids was covered by stratified columnar epithelium with 18–21 layers of nucleated cells, while the bulbar zone of these surfaces was covered by cubic multilayer epithelium with 6–11 non-keratinized layers of cells and with sparse goblet cells. In only lower eyelids, numerous lymphoid nodules, diffuse lymphocytes and high endothelial venules were observed. The superficial gland was an acinar complex which secreted mucous and contained plasma cells within the interlobular and interlobular connective tissue. The upper and lower branches of the third eyelid were the shape of a bent “caudal fin” and were composed of hyaline cartilage, and they contained conjunctiva associated lymphoid tissue (CALT). The deep gland was also an acinar complex producing a serous character and having numerous diffuse lymphocytes. The lacrimal gland was an acinar complex producing seromucous secretions and had numerous plasma cells located in the glandular interstitium. The results of our research indicate that the features of the anatomy of the eyelids and orbital region in the lowland tapir are also typical of the family Tapiridae, but also have features common to the families Equidae and Rhinocerotidae. We confirm the presence of poorly developed tarsal glands in both eyelids as well as presence of a palpebral part of the lacrimal gland in the upper eyelid, which is typical only to *Tapirus terrestris*.

## 1. Introduction

The lowland tapir (*Tapirus terrestris*), also commonly called the South American tapir, the Brazilian tapir or the Amazonian tapir, is the largest terrestrial autochthone herbivore in the Neotropical ecosystems [1,2]. The families Tapiridae and Rhinocerotidae belong to the Ceratomorpha suborder, while the family Equidae belongs to the Hippomorpha suborder, being part of the taxonomic order Perissodactyla, which is adapted for specialization of diet and running [3,4,5]. Tapirs used to occur in a very broad geographic range (North America, South America, Europa and Asia) [4]. They derive from an ancient group of animals related to primitive horses and rhinoceroses, represented from the late Paleocene to the present [4,6,7,8]. In the world, there are four Tapir species: Malayan tapir (*Tapirus indicus*) in Asia, and Baird’s tapir (*Tapirus bairdii*), mountain tapir (*Tapirus pinchaque*), and lowland tapir (*Tapirus terrestris*) in Central and South America, and these prefer wet and seasonally inundated areas, but also moist forests, savannas and grassland [2,6,9,10,11,12].

Tapirs for the most part are crepuscular or nocturnal, but their activity has mainly been described before sunrise and after sunset [5,6]. Tapirs have very good hearing, which is the dominant sense, but they have a poorly developed sense of sight [13,14,15,16,17].

Although cases of eye diseases among tapirs are not described often [6,15,18,19,20,21,22,23,24], the same is the case with morphological studies of the eyeball and accessory organs of the eye that are still unknown. Barongi (1997) reported that the most common problems with eye diseases in tapirs living in zoos occur in pools with a lack of shade [25]. He considers that the climate influences eye conditions in the tapir (Barongi, pers. comm) [25]. Ophthalmic cases in the horse and donkey are reported more frequently and to a lesser extent in rhinoceros [26,27,28,29,30,31,32,33,34,35,36,37,38,39,40,41,42,43,44]. The morphology of the eyeball and adnexa of the eye has been described more frequently in the family Equidae: donkeys, horses and the Rhinocerotidae family [45,46,47,48,49,50,51,52,53,54,55,56,57,58,59,60].

Because the lowland tapir is a species at risk of extinction, and also a frequent inhabitant of zoos around the world where veterinarians perform various ophthalmological procedures, it is therefore worth learning about the structure of the adnexa of the eye at the anatomical, histological and histochemical levels so that these doctors can provide the right treatment. Furthermore, our research aims to compare the morphology of the adnexa of the eye with those of other species included in Perissodactyla, to reveal similarities and differences among these animals.

## 2. Materials and Methods

**Figure 1:**
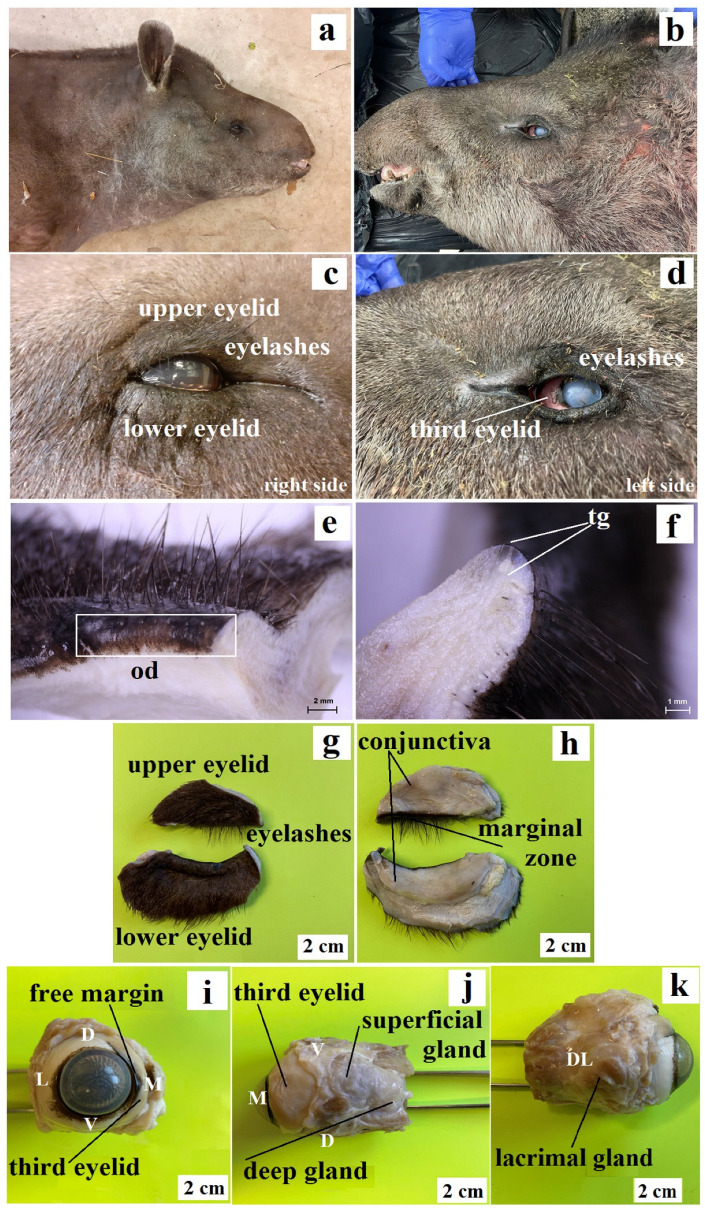
Photomacrographs of *Tapirus terrestris*. First female (**a**); second female (**b**); upper and lower eyelids with eyelashes, eyelid (**c**,**d**,**g**); tarsal gland duct openings (**e**); od, tarsal gland (**f**); tg, conjunctiva and marginal zone (**h**); third eyelid with free margin (**i**); third eyelid with superficial of the third eyelid and deep gland of the third eyelid (**j**); lacrimal gland (**k**). Eye angles: D, dorsal; V, ventral; L, lateral; M, medial, DL, dorsolateral.

Animals and tissue preparation. The chosen accessory organs of the eye (upper eyelids, lower eyelids, superficial gland of the third eyelid, third eyelid, deep gland of the third eyelid and lacrimal gland) were collected from two female adult lowland tapirs derived from the Wroclaw Zoological Garden (Poland). These animals was collected post mortem in 2019 and 2021 in the Division of the Animal Anatomy at Wroclaw University of Environmental and Life Sciences (Figure 1a,b). The first female was 25 years, 3 months and 27 days old (date of death—22 October 2019) and the second female was 31 years, 1 month and 2 days old (date of death—10 March 2021). The first female (named Sonia) was born in the Wroclaw ZOO (Poland). The second female (named Sabrina) was born in Rome ZOO (Italy). Veterinary medical history and post mortem ophthalmological examination excluded the presence of pathological changes within the orbit in the examined animals.

The length, width and thickness of the adnexa of the eye were measured using an electronic slide caliper with an accuracy of 0.1 mm (Stainless Hardened, Farnell, Poland). A Zeiss Stemi 2000-C stereoscopic microscope and Axio Vision 4.8 computer program (Carl Zeiss, Jena, Germany) were used for morphologic and morphometric assessment. The statistical analyses include mean and standard deviation (six random measurements).

The samples of the adnexa of the eye were fixed directly in 4% buffered formaldehyde for at least 72 h and next rinsed in running water for 24 h.

Then, the tissue was dehydrated using 75%, 96% and 100% solutions of ethanol in a vacuum tissue processor (ETP, RVG3, INTELSINT, Turin, Italy). The paraffin blocks were cut into sections of 5 μm on the Micron HM310 microtome (Boise, ID, USA). The following stains were performed: hematoxylin and eosin, Masson-Goldner trichrome, Movat pentachrome, mucicarmine, picro-Mallory trichrome, periodic acid-Schiff, alcian blue pH 1.0, alcian blue pH 2.5, alcian blue pH 2.5 PAS and Hale’s dialysed iron [61,62,63,64,65,66,67]. Next, tissue was analyzed using the Zeiss Axio Scope A1 light microscope (Carl Zeiss, Jena, Germany). All stains were performed in the Division of Histology of the Faculty of Veterinary Medicine at Wroclaw University of Environmental and Life Sciences. Anatomical and histological descriptions of the adnexa of the eye were based on NAV (2017) [68] and NAH (2017) [69].

## 3. Results

### 3.1. Macroscopic Morphology of the Eyelids and Orbital Glands

In the anterior palpebral margin of the upper eyelid, long, thick and fairly stiff eyelashes were observed, while they were thin, short and much less prominent in the lower eyelid (Figure 1c,d,g). Moreover, on the posterior palpebral margin, both eyelids had faintly visible tarsal glands (Figure 1e,f). Moreover, the presence of long boots of single sensory hairs (eyebrows) above the upper eyelid was noted. The palpebral conjunctiva was not pigmented, while the anterior palpebral margins had a brown color (Figure 1h). The mean size of the upper eyelid was 34.384 (±0.5) × 22.176 (±0.6) × 7.321 (±0.5) mm, while that of the lower eyelids was 38.795 (±0.4) × 18.08 (±0.2) × 9.917 (±0.3) mm.

The free margin of the third eyelid was gently pigmented (brow-black color) and was thin. The eyelids were similar to other animals in macroscopic anatomy and composed of an upper branch, lower branch and a crossbar. The crossbar was surrounded by a superficial gland of the third eyelid (Figure 1i,j) [27]. The upper and lower branches in the examined animals were similar to the bent “caudal fin”. The mean size of the lengths of the upper and lower branches together and the crossbar were 30.943 (±0.6) mm and 32.569 (±0.5) mm. The bulbar conjunctiva of the third eyelid did not show conjunctival crypts in the macroscopic image.

The superficial gland of the third eyelid was oval and light pink color (Figure 1j). The mean size was 16.153 (±0.5) × 17.756 (±0.5) × 9.955 (±0.2) mm. The deep gland of the third eyelid was oval in shape and had milky color (Figure 1j). The mean size was 15.87 (±0.6) × 13.976 (±0.3) × 8.195 (±0.3) mm. The lacrimal gland was close to triangular in shape and light pink in color (Figure 1k). The mean size was 24.592 (±0.6) × 20.028 (±0.6) × 8.815 (±0.2) mm. The location of the orbital glands and third eyelid was similar to that in other mammals [27,70,71].

### 3.2. Histological and Histochemical Analysis of the Eyelids and Orbital Glands

**Figure 2:**
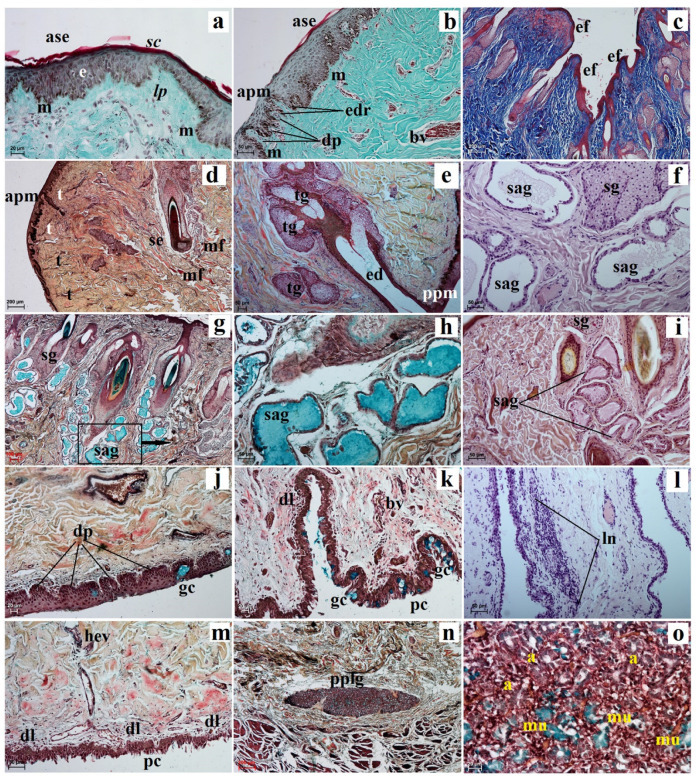
Photomicrographs showing histology of the *Tapirus terrestris* upper and lower eyelid. a—acini, apm—anterior palpebral margin, ase—anterior surface of eyelid, bv—blood vessels, e—epithelium, ed—excretory duct of the tarsal gland, edr—epidermal ridges, ef—eyelids folds, dl—diffuse lymphocytes, dp—dermal papillae, gc—goblet cells, hev—high endothelial venules, ln—lymphoid nodule, lp—lamina propria, m—melanocytes, mf—muscle fibres, mu—mucous units, pc—palpebral conjunctiva, pplg—palpebral part of the lacrimal gland, ppm—posterior palpebral margin, sag—simple alveolar gland, sc—stratum corneum, se—stroma of eyelid, sg—sebaceous glands, t—tarsus, tg—tarsal glands. Scale bars: (**c**,**d**,**n**) 200 µm; (**g**) 100 µm; (**b**,**e**,**f**,**h**,**i**,**l**,**m**) 50 µm; (**a**,**j**,**k**,**o**) 20 µm; (**a**,**b**) Masson-Goldner trichrome stain; (**c**) picro-Mallory trichrome stain; (**d**,**e**,**g**,**h**,**j**,**k,m**–**o**) Movat-pentachrome stain; (**f**,**l**) H&E stain; (**i**) mucicarmine stain.

**Figure 3:**
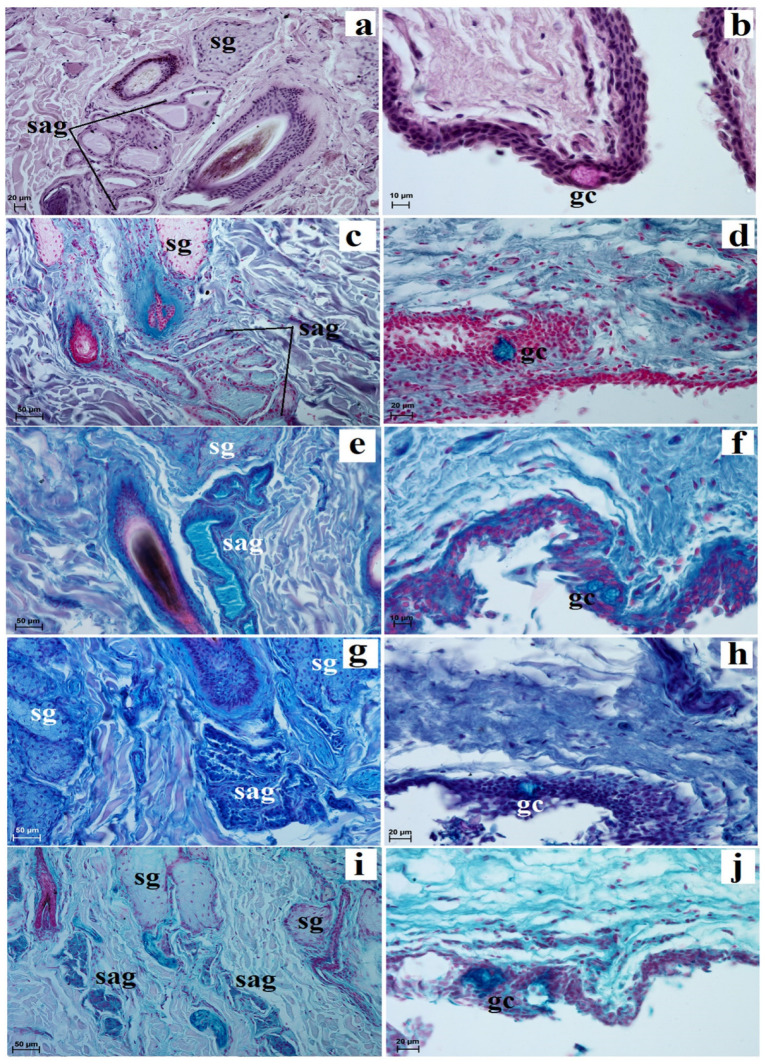
Photomicrographs showing histochemistry of the *Tapirus terrestris* upper and lower eyelid. gc—goblet cells, sag—simple alveolar gland, sg—sebaceous glands. Scale bars: (**c**,**e**,**g**,**i**) 50 µm; (**a**,**d**,**h**,**j**) 20 µm; (**b**,**f**) 10 µm; (**a**,**b**) PAS stain; (**c**,**d**) AB pH 1.0 stain; (**e**,**f**) AB pH 2.5 stain; (**g**,**h**) AB pH 2.5/PAS stain; (**i**,**j**) HDI stain.

The *facies anterior palpebrarum* was covered by stratified squamous epithelium composed of 11 to 13 layers of nucleated cells (Figure 2a). The superficial layer of epithelium was covered with a thin *stratum corneum* (Figure 2a), while the *stratum basale* contained a small number of melanocytes (Figure 2a). The junction between the epidermis and dermis featured irregular, small but pronounced dermal papillae and epidermal ridges (Figure 2b). The anterior surface of the both eyelids was characterized by the presence of numerous folds (Figure 2c). In both eyelids, no tarsal glands were found in the visual field. The upper and lower tarsus consisted of dense fibrous connective tissue (Figure 2d). The posterior palpebral margins of both eyelids were characterized by the presence of poorly developed tarsal glands, and directly below the tarsal plate, poorly developed tarsal glands were observed (Figure 2e). The stroma of both eyelids was made of dense, irregular connective tissue in which numerous and highly branched sebaceous glands and simple alveolar glands were arranged. There were delicately marked muscle fibers in the central part of the stroma and strongly marked muscle fibers around the *facies posterior palpebrarum* (Figure 2f,g). The simple alveolar glands were composed of a cubic monolayer epithelium and produced mucus (Figure 2h,i). The posterior surface of these eyelids consisted of the anterior palpebral margins and the palpebral conjunctiva (Figure 2b,d). The anterior palpebral margin was covered by stratified columnar epithelium with 18 to 21 layers of nucleated cells, with numerous melanin granules located only in the *stratum basale* (Figure 2a,b). The palpebral conjunctiva was covered by 6 to 11 non-keratinized layers of cells with goblet cells (Figure 2j). Numerous lymphoid nodules, diffuse lymphocytes and high endothelial venules were observed only in the lower eyelids (Figure 2k,m). The upper eyelids consisted of a palpebral part of the lacrimal gland with mucous units and serous acini (Figure 2n,o). A negative reaction was observed in the sebaceous glands and tarsal glands, and medium or strongly proteinaceous staining was seen in the simple alveolar glands (Figure 3a,c,d,g,i and Table 1). Strong staining was observed in the goblet cells in the palpebral conjunctiva (Figure 3a,d,f,h,j and Table 1).

**Figure 4:**
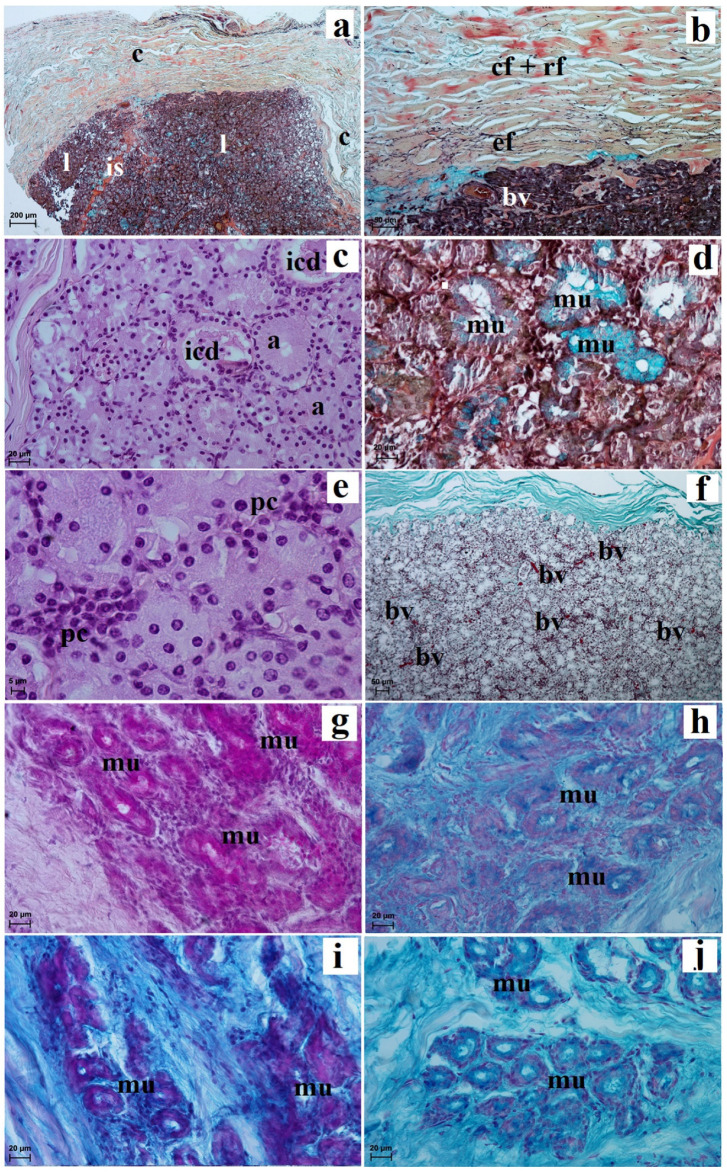
Photomicrographs showing histology and histochemistry of the *Tapirus terrestris* superficial gland of the third eyelid. a—acini, bv—blood vessels, c—capsula, cf + rf—collagen fibres and reticular fibres, dl—diffuse lymphocytes, ef—elastic fibres, icd—intercalated duct, is—interlobar septae, l—lobes. Scale bars: (**a**) 200 µm; (**b**,**f**) 50 µm; (**c**,**d**,**g**–**j**) 20 µm; (**e**) 5 µm. (**a**,**b**,**d**) Movat-pentachrome stain; (**c**,**e**) H&E stain; (**f**) Masson-Goldner stain; (**g**) PAS stain; (**h**) AB pH 2.5 stain; (**i**) AB pH 2.5/PAS stain; (**j**) HDI stain.

The superficial gland of the third eyelid was surrounded by a very thick connective tissue capsule, which was composed of thin and thick sparse interlobar septa, which divided this gland into big and middle lobes (Figure 4a). The glandular capsule was composed of dense connective tissue (Figure 4b). The secretory units had a small lumen composed of tall conical cells with eosinophilic cytoplasm, while the intercalated ducts were lined by a cuboidal epithelium (Figure 4c). The Movat-pentachrome stain showed the presence of mucous secretory units with a strong reaction (+++) (Figure 4d,e). Between the secretory units, interlobular and interlobular ducts, plasma cells and very numerous blood vessels were observed (Figure 4e,f). This was a multilobar acinar gland, and it histochemically produces a mucous-like secretion (Figure 4g–j and Table 1).

**Figure 5:**
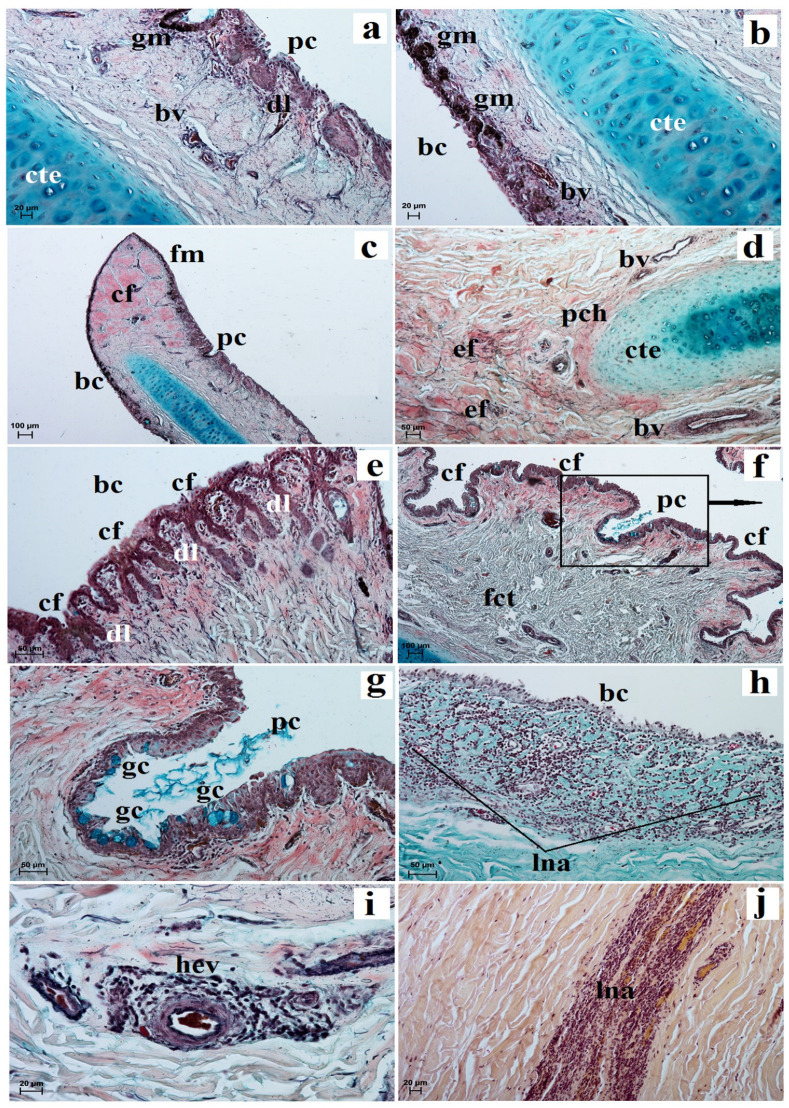
Photomicrographs showing histology of the *Tapirus terrestris* third eyelid. bc—bulbar conjunctiva, bv—blood vessels, cf—conjunctival fold, cte—cartilage of the third eyelid, dl—diffuse lymphocytes, ef—elastic fibres, fct—fibrous connective tissue, fm—free margin, gc—goblet cells, gm—granules of melanin, hev—high endothelial venules, lna—conjunctival lymph nodule, pc—palpebral conjunctiva, pch—perichondrium. Scale bars: (**c**,**f**) 100 µm; (**d**,**e**,**g**,**h**) 50 µm; (**a**,**b**,**i**,**j**) 20 µm. (**a**–**g**,**i**) Movat-pentachrome stain; (**h**) Masson-Goldner trichrome stain; (**j**) mucicarmine stain.

The palpebral conjunctiva of the third eyelid was covered by a non-keratinized cylindrical epithelium composed of 7 to 10–11 layers of nucleated cells. The bulbar conjunctiva contained a cubic multilayer epithelium consisting of 6 to 8–9 layers of cells (Figure 5a,b). The *stratum basale* epithelial cells in the palpebral and bulbar conjunctiva contained a large number of granules of melanin (Figure 5a,b). The free margin was composed of dense connective tissue with sparse blood vessels (Figure 5c). The surface cartilage was surrounded by thin layers of perichondrium composed of collagen, reticular and elastic fibers (Figure 5d). The cartilage of the third eyelid was composed of hyaline cartilage (Figure 5a,b,d). The bulbar conjunctiva was characterized by the presence of numerous small narrow conjunctival folds with numerous goblet cells, while the palpebral conjunctiva had a few large and wide conjunctival folds that also contained numerous goblet cells (Figure 5e–g). Within the conjunctival folds and stroma of these eyelids, diffuse lymphocytes with high endothelial venules and conjunctival lymph nodule aggregates were observed (Figure 5e,f,h–j).

**Figure 6:**
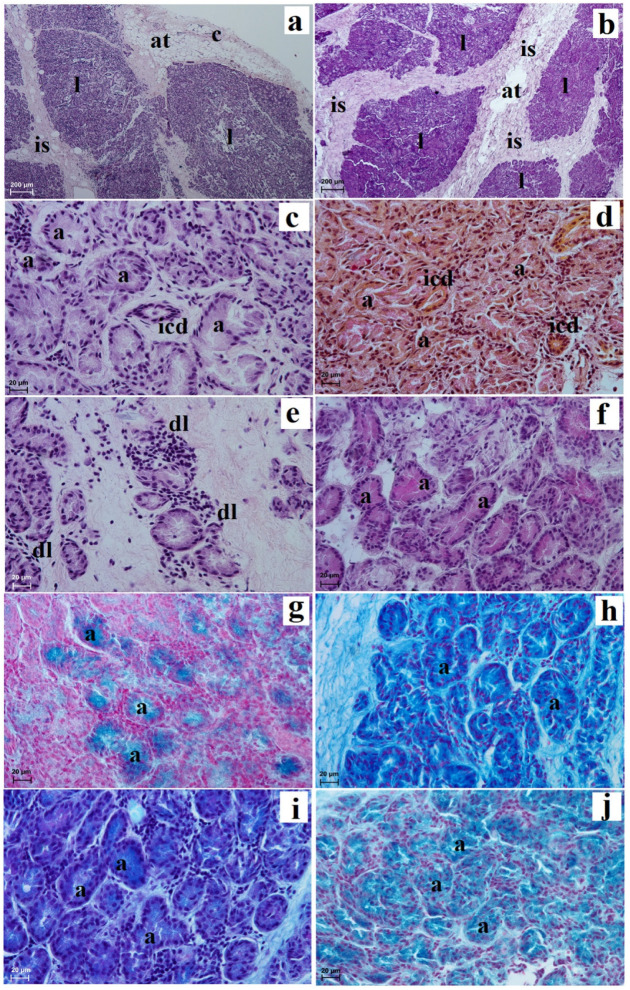
Photomicrographs showing histology and histochemistry of the *Tapirus terrestris* deep gland of the third eyelid. a—acini, at—adipose tissue, c—capsule, dl—diffuse lymphocytes, is—interlobar septa, l—lobes. Scale bars: (**a**,**b**) 200 µm; (**c**–**j**) 20 µm; (**a**–**c**,**e**) H&E stain; (**d**) mucicarmine stain; (**f**) PAS stain; (**g**) AB pH 1.0 stain; (**h**) AB pH 2.5 stain; (**i**) AB pH 2.5/PAS stain; (**j**) HDI stain.

The deep gland of the third eyelid was covered by a thick capsule which formed a thin and very thick interlobular septae, dividing the glandular stroma into small and big lobes (Figure 6a,b). In the capsule and intralobar septa, adipose tissue was observed (Figure 6a,b). The acini had a wide lumen and were composed of cuboidal cells with basophilic cytoplasm and a round nucleus lying in the basal part of the cell (Figure 6c). The intercalated ducts were composed of cuboidal epithelium with associated myoepithelial cells. The mucicarmine stain showed the presence of the secretory units with a negative reaction (−) (Figure 6d). The glandular interstitium characterized the numerous diffuse lymphocytes (Figure 6e). The deep gland was a multilobar complex acinar gland, and histochemically it is serous in character (Figure 6f–j and Table 1).

**Figure 7:**
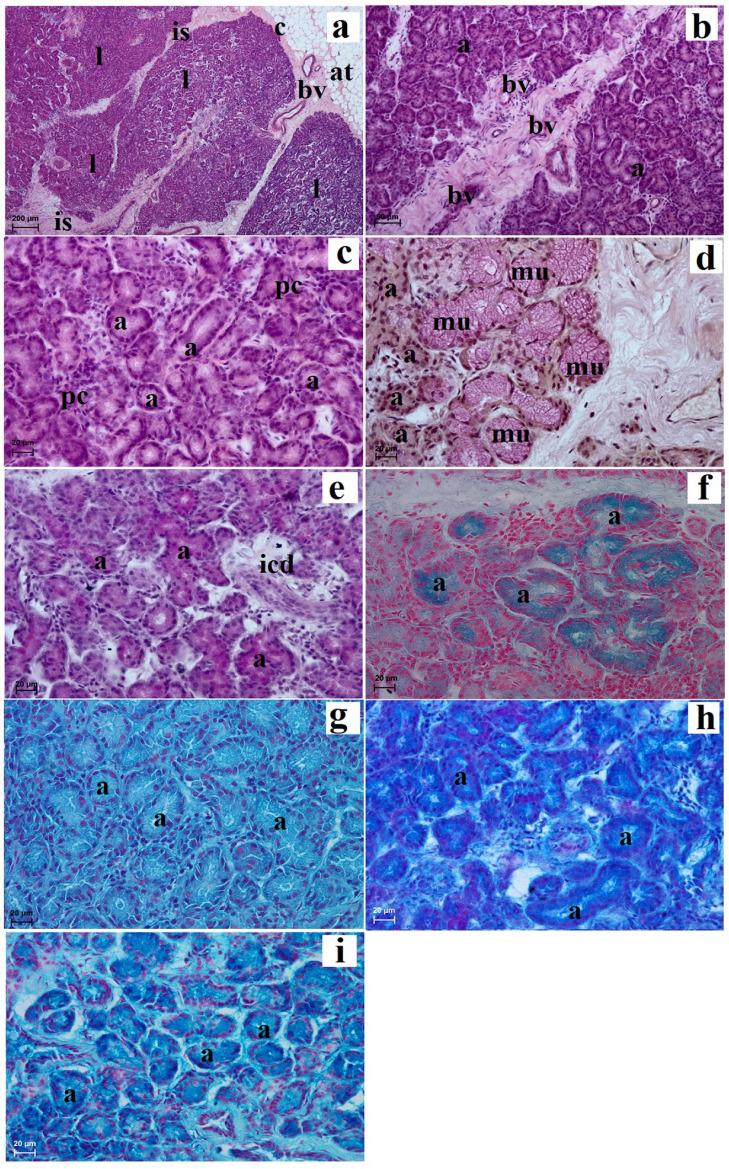
Photomicrographs showing histology and histochemistry of the *Tapirus terrestris* lacrimal gland. a—acini, at—adipose tissue, bv—blood vessels, c—capsule, icd—intercalated duct, is—interlobar septa, l—lobes, pc—plasma cells. Scale bars: (**a**) 200 µm; (**b**) 50 µm; (**c**–**i**) 20 µm; (**a**–**c**) H&E stain; (**d**) mucicarmine stain; (**e**) PAS stain; (**f**) AB pH 1.0 stain; (**g**) AB pH 2.5 stain; (**h**) AB pH 2.5/PAS stain; (**i**) HDI stain.

The lacrimal gland was surrounded by a thin connective tissue capsule containing numerous blood vessels which were surrounded by an extraperiorbital fat body (Figure 7a). A faintly marked connective tissue septum divides these glands into very large dominant lobes and less numerous small lobes (Figure 7a). In the intralobar septa, numerous blood vessels were observed (Figure 7b). The acini had a small lumen and were made of tall conical secretory cells with an eosinophilic cytoplasm with a round nucleus lying in the parabasal part of the cell (Figure 7c). The mucicarmine staining method showed the presence of few mucous units with a middle positive reaction (++) (Figure 7d). The numerous intercalated ducts were lined with a monolayer cubic epithelium (Figure 7e). In addition, numerous plasma cells in the glandular interstitium were observed (Figure 7c). The lacrimal gland had a multilobar acinar complex structure, and histochemically it had a seromucous nature (Figure 7e–i and Table 1).

## 4. Discussion

Tapirs and rhinos from the Ceratomorpha suborder are more closely related to each other than they are to horses and other Hippomorpha [72]. Unfortunately, there is a lack of studies concerning the exact accessory organs of eye anatomy and few studies in the field of veterinary ophthalmology in the family Tapiridae. We hope that the presented research results significantly expand the existing knowledge on the comparative anatomy of chosen adnexa of the eye in one of the four living families of Tapiridae representatives. In addition, we wanted to show whether there are features in the structure of these organs that are characteristic only for tapirs, common features, differences for closely related Tapiridae and Rhinocerotidae, or common features for the entire taxonomic order of Perissodactyla.

Macroscopic studies of both eyelids in two female lowland tapirs showed the presence of long, thick and fairly stiff eyelashes, while the lower eyelids were thin, short and much less prominent, as previously described for the family Rhinocerotidae [56,57,60]. Research by Pocock (1914) [73] in the made-on museum skins and mounted specimens only showed the presence of eyelashes in the upper eyelid. In the case of the equine and donkey, the prominent eyelashes are located on the upper eyelid, and the eyelashes provide tactile sensory perception [74]. There are no eyelashes on the lower eyelid, but fine hairs grow near the anterior palpebral margin [27,45]. Histologically, both eyelids in the lowland tapir were structurally similar to the horse, but our examined animals presented characteristic simple alveolar glands with mucous, but also poorly developed tarsal glands [27,72,73]. We suppose that the poor degree of development of the tarsal glands in the examined tapirs may result from the fact that there are additionally well-developed simple alveolar glands within the upper and lower eyelid and the presence of a palpebral part of the lacrimal gland and deep gland of the third eyelid. We hypothesize that these additional structures may cause a change in the proportions of the precorneal tear film, where the mucin layer may predominate. Consequently, it can increase the protection of the eyeball against harmful external factors due to the natural conditions in which tapirs live. Whether this is the case requires further post-mortem research on this animal species, not only in captivity but also in the wild, which we realize can be very difficult or even impossible. In addition, to demonstrate whether due to the presence of additional structures (deep gland of the third eyelid, palpebral part of the lacrimal part and simple alveolar gland in both eyelids) and poor development of the tarsal glands, there are changes in the rock of the individual three layers of the precorneal tear film, it may be useful for clinicians to evaluate in vivo ophthalmology results. Features common to tapirs and horses are the tarsal plate and the presence of sebaceous glands [27,75]. Another characteristic of the examined lowland tapir was the presence of the upper eyelid palpebral part of the lacrimal glands, which so far has not been demonstrated in other Perissodactyls, and is present, for example, in humans [76]. Our research also showed the presence of diffuse lymphocytes and specialized vessels defined as high endothelial venules in the lower eyelids structures of the CALT in the form of lymphoid follicles, which permits lymphocyte migration and exchange between ocular tissues and distant organs of the mucosal immune system [77,78]. In the domestic dog, age can influence antigenic stimulation [79]. Furthermore, Mastropasqua et al. (2017) [80] reported that any changes in the eyeball and adnexa relating to eye dysfunction caused by ocular surface diseases (OSDs) may have an impact not only on the correct vision process, but also on the comfort of the animal.

The free margin of this eyelid in the lowland tapirs was delicately pigmented, while in the horse was heavily pigmented [27,74]. According to Cooley (1972) [74], the degree of conjunctiva epithelial pigmentation is variable in breeds of horses. The upper and lower branches of the third eyelid in our examined animals were shaped like a bent “caudal fin”. However, according to Schlegel et al. (2001) [51], in the horse, the crossbar has a characteristic hook form, with a big incision from dorsal margin. Barnett et al. (2004) [27], Carastro (2004) [75] and Cooley (1992) [74] reported that in the horse the third eyelid is composed of T-shaped cartilage. In the histological study of the examined lowland tapirs, the cartilage of the third eyelid consisted of hyaline cartilage, while elastic cartilage was observed in the horse [51,70]. Moreover, in the examined lowland tapir, the presence of the conjunctival bulbar part of the conjunctival crypt, which occurs in the horse, was not macroscopically demonstrated [52]. Very numerous conjunctival folds present in the bulbar and palpebral conjunctiva in the lowland tapirs were only visible in the histological examination, which included diffuse lymphocytes with high endothelial venules and conjunctival lymph nodule aggregate as well as in horses [52,75]. Vallone et al. (2004) [52] reported that macroscopically, the third eyelid in equines contains conjunctival crypts that were not observed in our tapir. Moreover, both our lowland tapirs and equine conjunctiva of the third eyelid are characterized by a very large number of the goblet cells whose secretions are included in the inner-mucous layers of the precorneal tear film [27,81,82].

Studies in the examined lowland tapir showed that the superficial gland of the third eyelid, similar to the equine eyelid, was located in the medial angle of the eye [27,52,70,83]. Also, similar to in a horse, it was oval in shape and light pink in color [70]. Morphometric studies showed that the examined female superficial gland of the third eyelid was larger than the deep gland of the third eyelid. Since our research only featured two female specimens, we cannot refer to whether sexual dimorphism may affect the dimensions of the glands or other additional organs of the eye, but we suppose that such sexual dimorphism exists considering the research conducted on domestic dogs [84,85]. Histological analysis of the lowland tapirs showed that it is a branched acinar gland that produces mucous as compared to in equines where the superficial gland has a tubuloacinar structure and produces seromucous [27,52] or serous secretion [72,83], which contribute to the normal precorneal tear film [86].

Our research in the lowland tapir also showed the presence of a deep gland of the third eyelid, which was a complex acinar gland producing a serous-like secretion that does not occur in the *Equus* [27,70]. However, to confirm the presence or absence of this gland in Rhinocerotidae, anatomical and ophthalmological examination with the use of ultrasound should be performed. We suppose that the presence of simple alveolar glands in the eyelids producing mucous secretion and the presence of a deep gland of the third eyelid, which produces serous secretions, may be related to the fact that tapirs live in wet and marshy tropical forests.

In our study, the lacrimal gland of the lowland tapirs was located in the dorsolateral angle of the eye as in equine and donkeys [27,44,45,70,75,87]. These glands in the examined animals had a triangular shape approaching the oval similar to that of a donkey [87], and according to Alsafy (2010) [44], the shape of this gland is determined by its position. Morphometric analysis showed that this gland in the female lowland tapirs was much larger compared to the tested superficial gland and deep gland of the third eyelid. Moreover, when comparing the dimensions of the lacrimal gland in the examined females to the measurements made in donkeys and horses, this gland was significantly smaller in lowland tapirs [44,71,88,89,90]. Histological and histochemical studies have shown that this gland in the examined females had an acinar structure with a mucouserous nature, and in equine, this gland has a tubuloacinar structure [91] and whose secretion, as well as the superficial gland of the third eyelid, is part of the aqueous layer of the precorneal tear film [86].

## 5. Conclusions

In the structure of the eyelids and orbital glands, features typical of the *Tapirus terrestris* but also common between Equidae and Rhinocerotidae families were found, such as:

- presence of eyelashes in the anterior palpebral margin of both eyelids characterized by tapirs and Rhinocerotidae;
- presence of simple alveolar glands that produced a mucous secretion, characterized by *Tapirus terrestris*;
- presence of poor developed tarsal glands in the both eyelids;
- presence of the upper eyelid palpebral part of the lacrimal gland in only *Tapirus terrestris*;
- in the histological image in the conjunctival part of the third eyelid, presence of characteristic conjunctival folds with numerous diffuse lymphocytes, while in equine there are macroscopically visible conjunctival crypts;
- the presence of structures of the CALT organized form of lymphoid follicles, diffuse lymphocytes and high endothelial venules only in lower eyelids;
- the upper and lower branches of the third eyelid appeared in the shape of a curved “caudal fin”, while in the equine they could be T-shaped or in hook form;
- the third eyelid composed of hyaline cartilage in *Tapirus terrestris* and in horse elastic cartilage;
- the presence of CALT in the conjunctival part of the third eyelid in the lowland tapir as well as in equine;
- the superficial gland of the third eyelid in the lowland tapir is a branched complex alveolar gland that produces mucus, while in equine these glands have tubuloacinar structure and produce seromucus secretion;
- presence of only a deep gland of the third eyelid in the *Tapirus terrestris*;
- the lacrimal gland in the lowland tapir and equine is an acinar gland of mucoserous nature.

## Figures and Tables

**Table 1:** Histochemical examination of the upper and lower eyelids, superficial of the third eyelid, deep gland of the third eyelid and lacrimal gland in the two female lowland tapirs.

	Simple Alveolar Glands	Sebaceous Glands	Tarsal Glands	Goblet Cells in The Upper and Lower Eyelids	Superficial Gland of The Third Eyelid	Deep Gland of The Third Eyelid	Lacrimal Gland
PAS	−	−	−	+++	++/+++	−/+	++
AB pH 1.0	++	−	−	+++	−	+++	dominant + and sparse ++/+++
AB pH 2.5	+++	−	−	+++	++/+++	+++	+++
AB pH 2.5 PAS	+++ (blue color)	−	−	+++ (blue color)	++ (dominant magenta color)	+++ (blue color)	dominant +++ (blue color) and sparse +++ (magenta color)
HDI	+++	−	−	+++	+	+++	+++

The histochemical evaluation of the examined structures (eyelids and orbital glands) was performed according to Spicer and Henson [67]. − negative reaction, + weak positive reaction, ++ middle positive reaction, +++ strong positive reaction.

## Data Availability

Material available upon request to the corresponding authors.
